# Characterizing an engineered carotenoid-producing yeast as an anti-stress chassis for building cell factories

**DOI:** 10.1186/s12934-019-1205-y

**Published:** 2019-09-10

**Authors:** Hsien-Lin Liu, Jui-Jen Chang, Caroline Thia, Yu-Ju Lin, Shou-Chen Lo, Chieh-Chen Huang, Wen-Hsiung Li

**Affiliations:** 1 Ph.D. Program in Microbial Genomics, National Chung Hsing University and Academia Sinica, Taipei, Taiwan; 2 Department of Medical Research, China Medical University Hospital, No. 91 Hsueh-Shih Road, Taichung, 402 Taiwan; 3 Biodiversity Research Center, Academia Sinica, 128 Academia Road, Sec. 2, Nankang, Taipei 115 Taiwan; 4 Department of Life Sciences, National Chung Hsing University, No. 250, Kuo Kuang Rd, Taichung, 402 Taiwan; 5 Innovation and Development Center of Sustainable Agriculture, National Chung Hsing University, No. 145, Xingda Rd, South Dist, Taichung, 402 Taiwan; 6 Department of Ecology and Evolution, University of Chicago, Chicago, IL 60637 USA

**Keywords:** Carotenoids, Toxins, Bio-ethanol, Anti-stress, 10-deacetylbaccatin III, Anti-stress, Biorefinery

## Abstract

**Background:**

A microorganism engineered for non-native tasks may suffer stresses it never met before. Therefore, we examined whether a *Kluyveromyces marxianus* strain engineered with a carotenoid biosynthesis pathway can serve as an anti-stress chassis for building cell factories.

**Results:**

Carotenoids, a family of antioxidants, are valuable natural products with high commercial potential. We showed that the free radical removal ability of carotenoids can confer the engineered host with a higher tolerance to ethanol, so that it can produce more bio-ethanol than the wild type. Moreover, we found that this engineered strain has improved tolerance to other toxic effects including furfurals, heavy metals such as arsenate (biomass contaminant) and isobutanol (end product). Furthermore, the enhanced ethanol tolerance of the host can be applied to bioconversion of a natural medicine that needs to use ethanol as the delivery solvent of hydrophobic precursors. The result suggested that the engineered yeast showed enhanced tolerance to ethanol-dissolved hydrophobic 10-deacetylbaccatin III, which is considered a sustainable precursor for paclitaxel (taxol) bioconversion.

**Conclusions:**

The stress tolerances of the engineered yeast strain showed tolerance to several toxins, so it may serve as a chassis for cell factories to produce target products, and the co-production of carotenoids may make the biorefinary more cost-effective.

## Background

Due to the increasing demand of alternative fuel and biopharmaceuticals, people are looking for reliable and sustainable ways to produce various bio-products. Synthetic biology, which can be used to design and integrate new biological functions into a cell, provides a powerful way to engineer a microbe for a sustainable bio-industry. Its broad applications can incorporate desirable bio-processes into a designer host to convert biomass to valuable bio-products such as biofuels [1]. Moreover, producing multiple bio-products simultaneously in a single microbe, such as co-production of biofuel and valuable natural products, can achieve competitive advantages for a multipurpose biorefinery [2, 3]. However, the toxic effects from precursors, intermediate products or end products can cause a physiological imbalance to the host. Exposure to xenobiotics may lead to an increase of reactive oxygen species (ROS) and free radicals intracellularly, which can cause cellular damages [4, 5]. The cellular membrane is an important boundary to protect cells from external stresses, but excessive free radicals can attack the membrane by lipid peroxidation. Previously, metallothioneins, which are the membrane-targeted antioxidative proteins, were demonstrated to improve the cell tolerance against *n*-butanol by scavenging intracellular or extracellular ROS [6, 7]. Furthermore, n-butanol production was increased via co-expression of metallothioneins [8]. The results implied that the ROS scavenging capacity of the host cell is important for cell factory development.

In this study, the yeast *Kluyveromyces marxianus* was used as the host because it has several desirable characteristics for industrial applications. First, *K. marxianus* is a Crabtree-negative yeast that exhibits enhanced biomass production when supplementing with excessive glucose. Second, *K. marxianus* is capable of fermentation at a broad range of temperatures (25 to ~ 45 °C). Third, *K. marxianus* can utilize various carbon sources, including lactose, xylose, arabinose, cellobiose, and inulin. Finally, *K. marxianus* is GRAS (generally regarded as safe) and QPS (qualified presumption of safe) and has been widely employed in various biotechnological applications and food industry [9].

In our previous study, a carotenoid biosynthesis pathway was integrated into *K. marxianus* [10]. Carotenoids are superior antioxidants, which have an ability to neutralize singlet oxygen and to protect cellular membranes from ultraviolet (UV) light and toxic oxidative stresses [11]. Moreover, carotenoids have been used in the prevention of various human diseases [12, 13] and may act as value-added products.

In this study, we tested if our carotenoid-producing *K. marxianu* strains can tolerate toxic compounds that may appear in biofuel production. As consolidated bioprocessing (CBP) provides a simple way to integrate biomass hydrolysis and fermentation in one process, furfurals are often generated from lignocellulosic biomass hydrolysis and inhibit yeast growth. In addition, heavy metals may be present when utilizing biomass harvested from polluted environment. Increasing the tolerance of the host to heavy metals may enhance its ability to utilize biomass from phytoremediation. Phytoremediation may provide a cost-effective way to cleanup heavy metals in contaminated soil and water by plants, and the biomass can then be a source for renewable energy and bio-products [14]. Therefore, tolerance assays were conducted to evaluate the potential of carotenoids producing strain for biofuel production that may face ethanol, furfurals and heavy metals. In addition, we also tested the tolerance of the carotenoid-producing strain to ethanol dissolved with 10-deacetylbaccatin III, which is a precursor to paclitaxel, an anti-cancer medicine. Thus, this study explored the potential of using a carotenoid-producing strain as a chassis for constructing multipurpose cell factories.

## Results

### Characterization of two engineered strains

Our carotenoid-biosynthesis pathway includes the truncated 3-hydroxy-3-methylglutaryl -coenzyme A reductase (*tHMG1* gene) from *Kluyveromyces marxianus*, the geranylgeranyl pyrophosphate synthase (*crtE* gene) from *Xanthophyllomyces dendrorhous*, the *crtYB* gene (phytoene synthase/lycopene cyclase) of *X. dendrorhous*, the *crtI* gene (phytoene desaturase) of *X. dendrorhous*, the β-carotene ketolase (*bkt* gene) from *Chlamydomonas reinhardtii,* and the β-carotene hydroxylase (*chyb* gene) from *Chlorella zofingiensis*. In a previous study [10], the synthetic biology tool PGASO (Promoter-based Gene Assembly and Simultaneous Overexpression) [15] was employed to integrated the pathway into the host genome, and the seven gene cassettes (promoter-gene-terminator) including KlPLac4–*crtI*–KlTTLac4, ScPGapDH–crtE–ScTTGap, ScPGK–*chyb*–ScTTPGK, KlPGapDH–*kanMX*–ScTTGap, KlPGK–*bkt*–ScTTPGK, KlPADHI–*crtYB*–ScTTGap, and ScPADHI–*tHMG*–ScTTADHI, were co-transformed into the yeast host, *K. marxianus*. As the colony color was correlated with the carotenoids amount produced, one of the light red colonies was selected and denoted as Cz5 strain and the reddest colony was selected and denoted as the Cz30 strain.

**Fig. 1:**
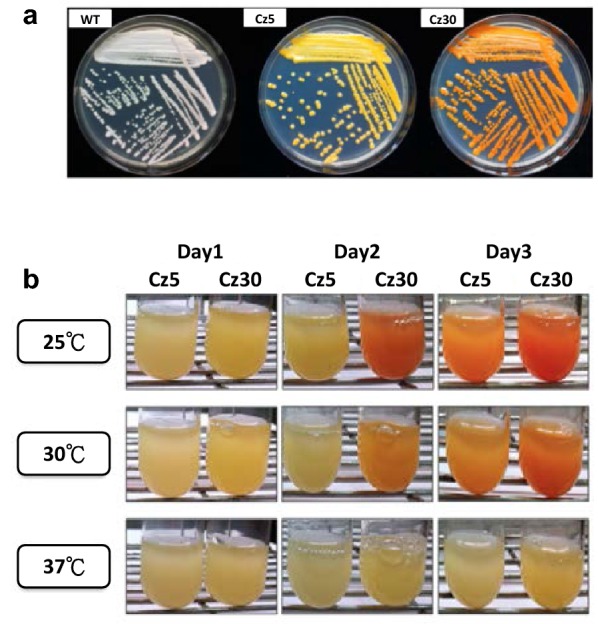
The carotenoid profiles of Cz5 and Cz30. **a** Different colors of the WT, Cz5, and Cz30 strains. **b** Growths of WT, Cz5, and Cz30 at 25 °C, 30 °C and 37 °C. The red color intensity indicates the conversion efficiency of carotenoids

In this study, we characterized the two engineered strains by color observation, transcription measurement, and metabolite analysis. Compared to the wild type (WT) strain, the Cz30 strain exhibited stronger red color than Cz5, while WT did not show red color (Fig. 1a). The yellow (Cz5) and red orange color (Cz30) implies the existence of carotenoids. The gene expression profiles of Cz5 and Cz30 at different growth temperatures (25 °C, 30 °C, and 37 °C) were examined by quantitative reverse transcription PCR (RT-qPCR). Since phytoene desaturase (encoded by *crtI*) and β-carotene ketolase (encoded by *BKT*) are the crucial enzymes in the production of 3S, 3′S-astaxanthin, two stronger promoters, pLac4 and pKlPGK, were used to drive these two genes. Accordingly, the RT-qPCR data indicated that the expression levels of the *CrtI* and *CrBKT* genes were higher than those of the other genes (Fig. 1b). Furthermore, all carotenoid-biosynthesis pathway genes of Cz30 showed higher expression levels than those of Cz5. Among the three growth temperatures (25 °C, 30 °C and 37 °C), the strongest color was found at 25 °C (Additional file 1: Fig. S1). The carotenoids profiles of Cz5 and Cz30 were further verified by HPLC spectrometry under UV460 nm. Carotenoids were detected in both Cz5 and Cz30, but not in WT (Additional file 1: Fig. S2). The total amount of carotenoids of Cz5 was ~ 137.2 µg/g [10], while that of Cz30 was ~ 250.5 µg/g. The data indicated that the Cz30 strain produced higher amounts of carotenoids than Cz5, and was chosen for anti-stress assays.

### Enhancement of free-radical scavenging capacity and cell protection ability

**Fig. 2:**
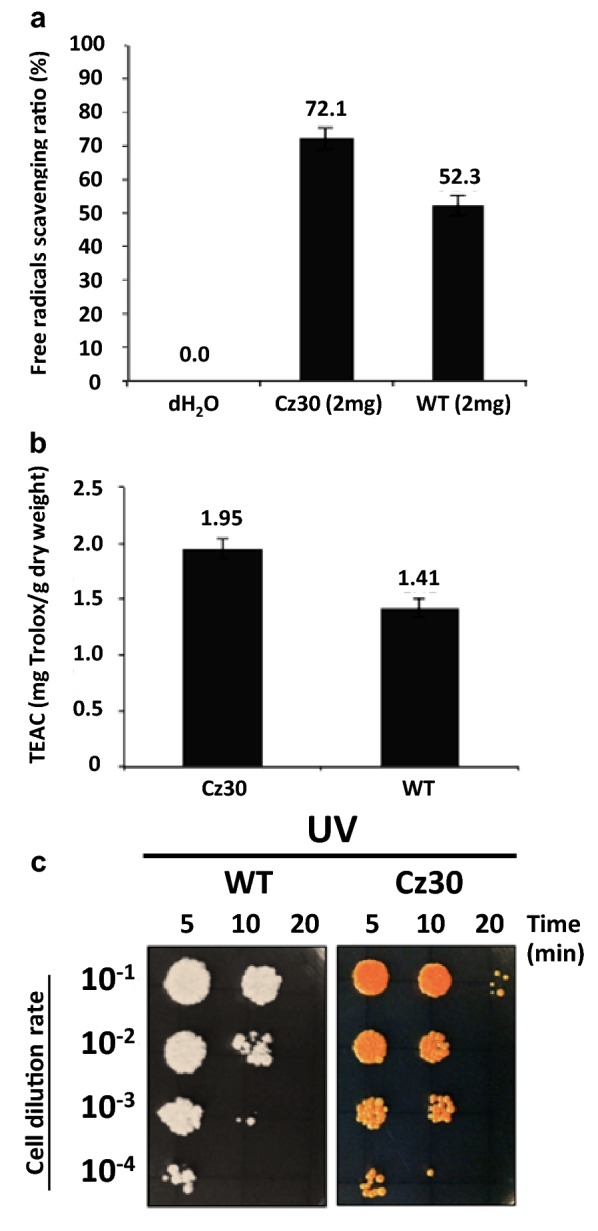
Free-radical scavenging capacity and cell survival under UV exposure. **a** The histogram of free radical scavenging ratios for Cz30. The ratio is determined by antioxidant capacity assay using ABTS. **b** The histogram of Trolox equivalent antioxidant. **c** The survival assay showing the colonies of WT and Cz30 with serial dilution after different titer exposures of UV. The data represent the mean ± SD (n = 3)

To determine the free radical scavenging capacity, the crude extract of Cz30 was used to evaluate the antioxidant ability using 2,2′-azino-bis (3-ethylbenzothiazoline-6-sulphonic acid) (ABTS) reaction and Trolox Equivalent Antioxidant Capacity (TEAC) assay. The extract of Cz30 showed a higher free radical scavenging capacity (72.1%) than that of WT (52.3%) by ABTS per 2 mg dry cell weight (Fig. 2a). For the TEAC assay, the antioxidant capacity of the Cz30 extract per gram dry cell weight was 1.95 mg of Trolox, while that of the WT extract was only 1.41 mg of Trolox (Fig. 2b). Thus, the carotenoids of the Cz30 strain might can increase ~ 38% free radicals scavenging capacity in yeast host.

To test the ability of carotenoids to prevent the cellular damage from UV light, the engineered strains were exposed to UV radiation for 5, 10, or 20 min. The survival rate was measured by the colony number counting. Cz30 showed a better survival rate than WT (Fig. 2c). Apparently, the antioxidant activity of carotenoids reduced the mortality of Cz30 from UV damage.

### Improving alcohol production

**Fig. 3:**
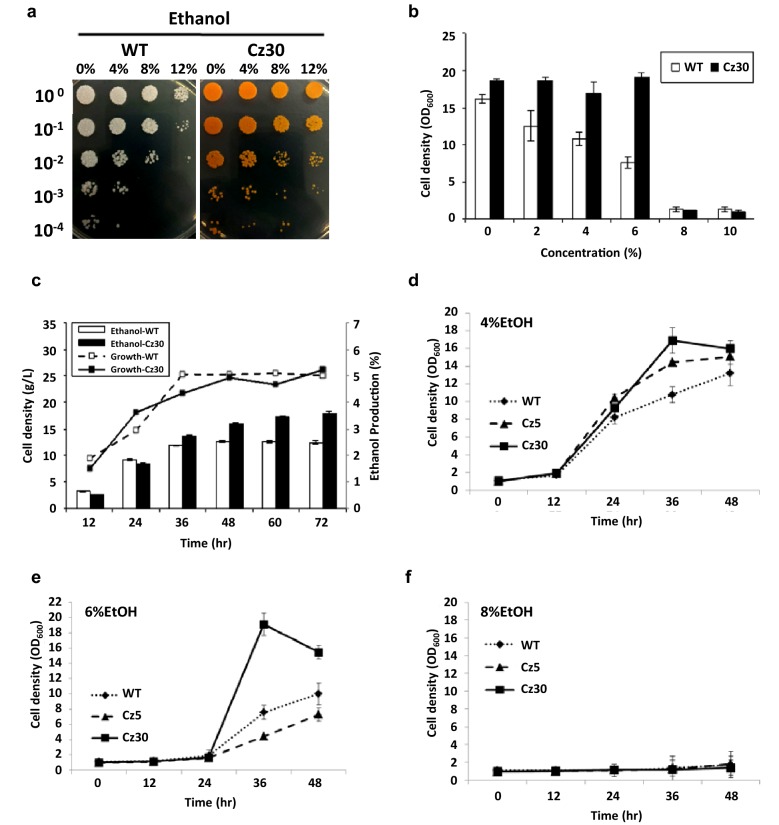
Alcohol tolerance and production of WT, Cz30 and Cz5. **a** The survival assay. The colonies of WT and Cz30 were exposed to 0%, 4%, 8% and 12% ethanol concentrations. **b** The growth assay. The cell densities of WT and Cz30 strains treated with different ethanol concentrations were measured at 36 h. Cz30 showed better growth than WT at 2%, 4% and 6% ethanol concentrations. **c** Cell growth and ethanol production of WT and Cz30 strains. The left y axis represents the cells density, the right y axis represents the ethanol production, and the x axis represents the time. Cz30 showed a higher ethanol production than WT. **d**–**f** WT, Cz5, and Cz30 were exposed to 4%, 6%, and 8% ethanol for 48 h in growth assay. Cz30 exhibited higher tolerance than Cz5 and WT at 4% and 6% ethanol. Cz5 exhibited better tolerance than WT at 4%, but not at 6%. WT, Cz5, and Cz30 did not grow at 8% ethanol at the initial cell density of 1.0 OD_600nm_. The data represent the mean ± SD (n = 3)

In the survival assay, the serial dilution testing showed that Cz30 has a better survival rate than WT in different concentrations of ethanol stress (Fig. 3a). Figure 3b shows that at 2, 4, or 6% ethanol, the cell growth of WT was significantly more strongly repressed by ethanol than that of Cz30. Moreover, Cz30 produced more ethanol (3.5%) compared to WT (2.5%) after 72 h (Fig. 3c). Figure 3d–f showed the growth curves of different strains. These data indicated that carotenoids might protect the host from the damage by ethanol during the fermentation process and improved the ethanol production. It also showed that Cz30 can be engineered to convert biomass to valuable carotenoids and ethanol simultaneously.

### Improvement of stress tolerance

**Fig. 4:**
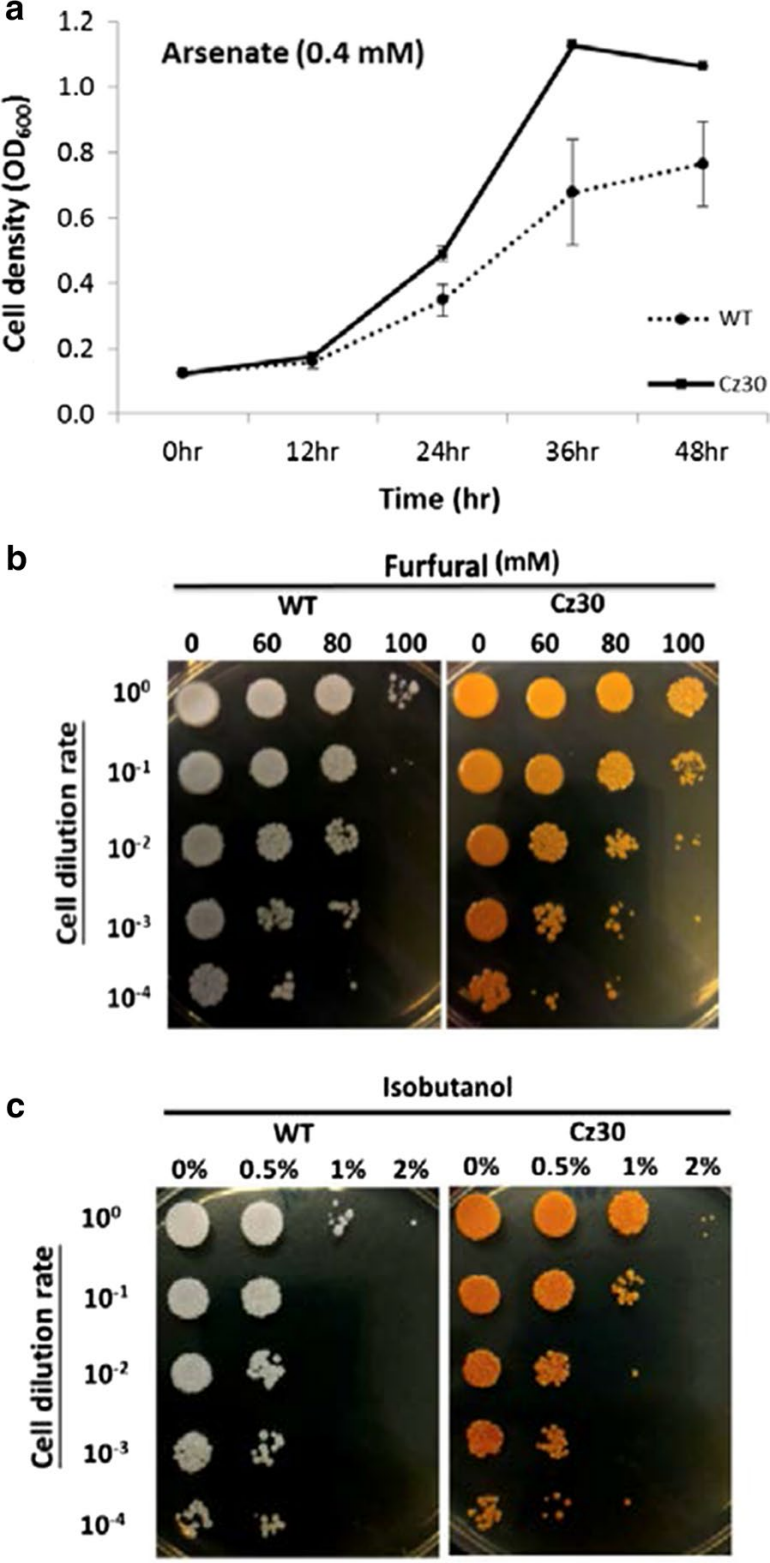
Growth assays under assenate, furfural and isobutanol. Cz30 grew better than WT under 0.4 mM arsenate (**a**). The serial dilutions after various titer exposures to furfural (**b**) and isobutanol (**c**) for 24 h showed a better survival rate for Cz30 than WT. The data represent the mean ± SD (n = 3)

Furfural and heavy metal arsenate (As[V], AsO_4_^3−^) can cause an oxidative stress in yeast [16, 17]. Furthermore, biofuels, such as isobutanol, can also cause stresses to yeast cells. The engineered strains were therefore tested for its ability to tolerate arsenate, furfural, and isobutanol. Compared to WT, Cz30 showed better tolerances to arsenate, furfural, and isubutanol (Fig. 4a–c). Hence, the production of carotenoids might protect Cz30 from various toxins.

### Improved tolerance of Cz30 to 10-deacetyl baccatin III

**Fig. 5:**
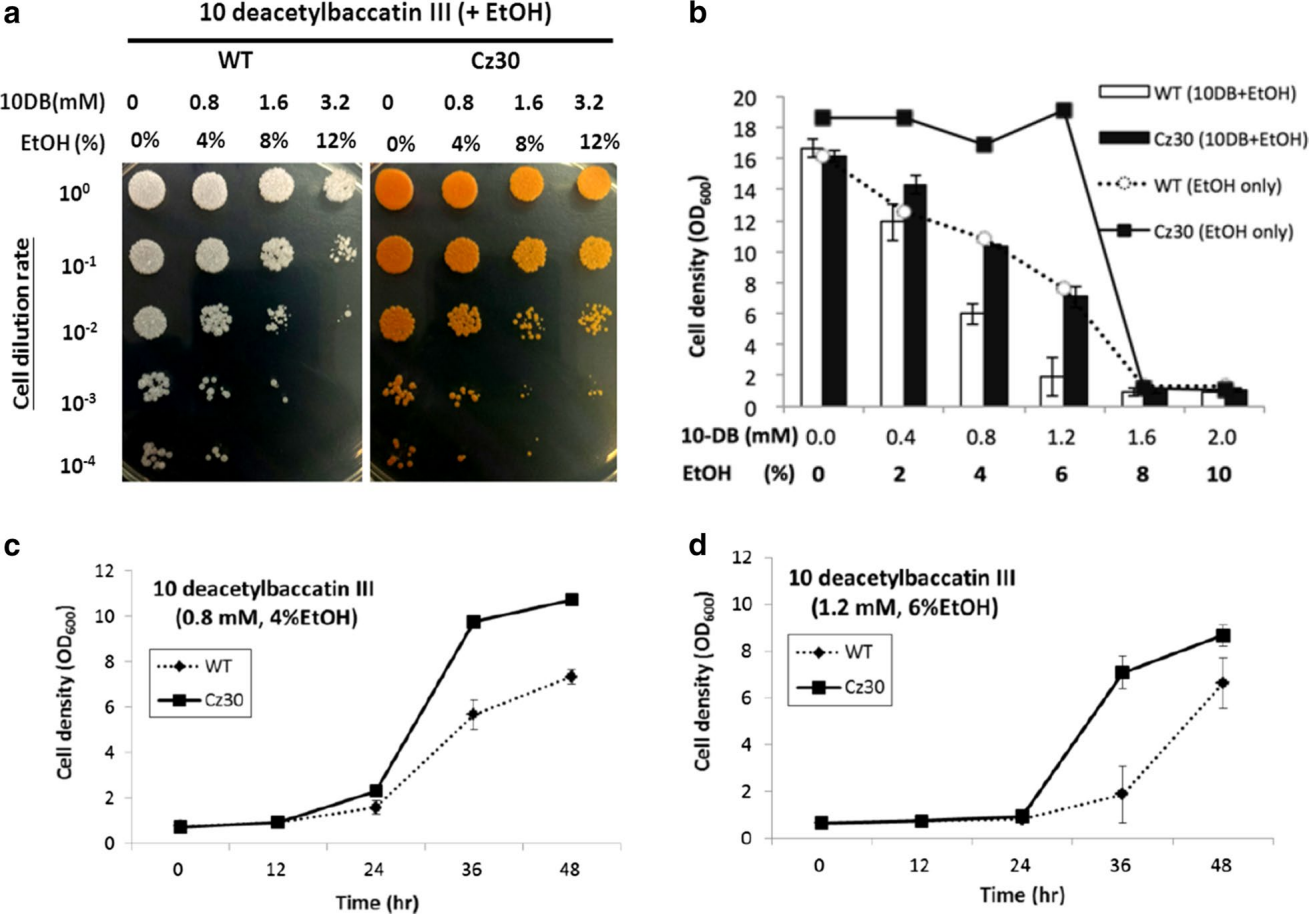
Survival assays under 10-deacetylbaccatin III and ethanol. **a** The survival of the colonies of WT and Cz30 strains under 10 deacetylbaccatin III (10-DB III) and ethanol. **b** The cell densities of WT and Cz30 strains after growth for 36 h in various concentrations of ethanol mixed with/without 10-DB III. **c**, **d** The cell growth assays under 0.8 mM 10-DB III (+4% EtOH) and 1.2 mM 10-DB III (+6% EtOH), respectively. The data represent the mean ± SD (n = 3)

The metabolite baccatin III is a crucial precursor for semi-synthesis of paclitaxel and its derivatives. The metabolite10-deacetyl baccatin III (10-DB III) is the natural precursor of baccatin III, and it has a high concentration (0.1%) in needle extracts of the common ornamental yew (*Taxus baccata*) [18], and therefore has been considered a cost-efficient and eco-friendly source. However, ethanol is an important solvent for dissolving this hydrophobic precursor. Due to the saturation solubility of 10-DB III in ethanol (20 mM), the ethanol tolerance became a limitation of substrate supplementation, and the improvement of ethanol tolerance could be helpful to overcome this obstacle. Hence, the tolerance of Cz30 was analyzed by treatment of ethanol-dissolved 10-deacetyl baccatin III. In survival assays, WT and Cz30 were exposed to 0, 0.8, 1.6 or 3.2 mM of 10-deacetyl baccatin III that were dissolved in 0, 4, 8 or 12% ethanol for 24 h, and subsequently inoculated into the YPG plate with a series dilution. The result showed that Cz30 had a better survival rate than WT (Fig. 5a). It was also subjected to the growth assay in YPG medium with different initial concentrations of ethanol supplemented with/without 10-DB III. The Cz30 showed better growth in ethanol supplemented with or without 10-DB III than WT (Fig. 5b). These results were supported by the growth curve assay under 0.8 mM of 10-deacetylbaccatin III with 4% ethanol and 1.2 mM of 10-deacetylbaccatin III with 6% ethanol (Fig. 5c, d). These data suggested that the carotenoids can protect the host cell from the damage by 10-deacetylbaccatin III. Thus, carotenoid-producing yeast strains can help the conversion of 10-deacetylbaccatin III to paclitaxel or its derivatives.

## Discussion

Simultaneously production of multiple bio-products with a good biorefinery approach may reduce cost. However, the host may confront more stresses. The tolerance to multiple stresses and/or toxins is a key challenge to improve the performance of bioconversion. Carotenoids are natural antioxidants that can help tolerate stresses and provide benefits to human health. This concept has here been shown by constructing a carotenoid-production yeast. By using the PGASO method, the target genes were integrated into the genome by homologous recombination that was targeted to the region of Lac4 promoter. The homologous sequence was modified from the Lac4 promoter, so that the integration did not disrupt the landing site but could create one more copy of the Lac4 promoter for further integration. This characteristic provided an opportunity for simultaneous multiple-integrations. Thus, different transgenic lines may carry different copy numbers of the transgenes. Based on the color, we screened strains with higher levels of carotenoids productivity in this study. CZ30 showed a stronger red color than Cz5 and indeed produced more carotenoids than Cz5. Though the transcriptional levels were higher at 30°Cthan at 25 °C, the carotenoid amount was higher at 25 °C. The reason is unclear, but carotenoids productivities may depend on not only the expression levels of carotenoid-biosynthesis genes, but also reaction dynamics between enzymes and substrates, which could be temperature dependent. According to HPLC profile, some new peaks were found and may belong to carotenoids family or carotenoids derivatives for further characterization (Additional file 1: figure S2).

Biorefinery provides a way to efficiently convert renewable biomass to sustainable products, such as biofuels, biochemicals, and biodegradable materials. Plant biomass is considered an abundant resource. However, to facilitate the bioconversion of biomass, pretreatment processes are conducted for lignocellulose destruction, which release various toxic substances and stresses, such as furfurals, which reduce the host performance of bio-alcohol fermentation [16]. We showed that an engineered carotenoid-producing *K. marxianus* improved the ability to scavenge free radicals and tolerance to UV damage, furfurals, and ethanol. The enhancement of ethanol tolerance also increased the production of ethanol. In a previous study, *K. marxianus* showed higher tolerance to harsh environment including furfural stress than *S. cerevisiae* and could be applied to pretreated lignocellulose [9]. Our study supported the potential application of carotenoid producing *K. marxianus* to biofuel production. It also proved the concept of biorefinary to produce ethanol and carotenoid simultaneously. Compared to common industrial yeasts including *S. cerevisiae* and *Pichia kluyveri, K. marxianus* can produce higher branched alcohol, such as isobutanol. The tolerance to isobutanol could make carotenoid producing *K. marxianus* more competitive to produce this next generation biofuel. Furthermore, the tolerance to heavy metal provides an attractive way to utilize phytoremediation biomass from contaminated areas. Improvement of cell tolerance by carotenoids increased the potential of multiple-product conversion and multiple resource utilization.

Carbon flux diversion is an inevitable issue when constructing a host to produce multiple products. In general, minimizing flux diversion of byproducts should be made for maximizing the main production. However, as the tolerance may also enhance the productivity or bio-conversion efficiency, it really depends on what kind of product that a cell factory is intended to produce. As we focused on the protection effect at this stage, we used Cz30 because it yielded 250.5 µg/g of carotenoids whereas Cz5 only yielded 137.2 µg/g. In fact, we found that Cz30 was more ethanol tolerant than Cz5 (Fig. 3d–f).

Natural secondary metabolites have broad pharmaceutical applications, but the yields are usually very low. In order to obtain a sufficient amount of compounds, chemical semi-synthesis provides a way to convert abundant precursors to the valuable products. However, the chemical process often incurs laborious manipulations and organic pollution. Bio-based production through engineered microorganisms provides a sustainable, reliable and efficient way for green production. To convert natural abundant intermediates to functional products could be faster than synthesis from central carbon flux. However, solvent plays a key role to deliver the hydrophobic intermediate to bioprocess, and the cell tolerance to solvent could be a limitation to bioconversion. For instance, paclitaxel has been used for cancer therapy for a long time and bioconversion is thought to be an efficient way for mass production of paclitaxel from abundant 10-deactylbaccatin III. Our carotenoid-producing strain not only improved the tolerance to ethanol but also to the paclitaxel precursor 10-deactylbaccatin III. It could be applied for improving bio-ethanol production and also for paclitaxel bioconversion. This discovery could be potentially applied to paclitaxel biopharmaceuticals industry.

## Conclusions

The engineered carotenoids-producing strain Cz30 showed an enhanced survival rate under the stresses of different toxins such as furfural, arsenate, ethanol, and isobutanol. In addition, the increased tolerance to ethanol allowed the host to deliver more 10-deacetylbaccatin III into the paclitaxel bioconversion process. Thus, our engineered strain Cz30 has great potential to serve as a chassis cell for bio-refinery.

## Methods

### RT-qPCR quantification

The RNA was purified by HiQ-Column 12 automated DNA/RNA Purification System (Protech, Taiwan) with an AccuPure Yeast RNA mini kit (AccuBioMed, Taiwan). SuperScript™ II Reverse Transcriptase (Invitrogen, USA) was used to convert RNA to cDNA. KAPA ™ PROBE FAST qPCR Kit (KAPAbiosystems, USA) and LightCycler 480 (Roche, USA) were conducted for qPCR analysis. The designer UPL (Universal ProbeLibrary, Roche) primer was shown in Table 1, and *Alg9* was used as a reference gene.

**Table 1:** UPL primer sets were used to measure relative quantification of each gene by qRT-PCR

Primer name	Sequence
crtE-UPL#1-F	CGAGATGCTTTCCCTCCATA
crtE-UPL#1-R	TTCGCTAGGACACGTCAGACT
crtI-UPL#155-F	CCGATCCTTCCTTTTACGTG
crtI-UPL#155-R	CGGCACAAGAATGACGATAG
crtYB-UPL#34-F	CACTGATCTTATCTTTCCCTTATCG
crtYB-UPL#34-R	GTGGTCTCGATAGGCGTCTT
tHMG-UPL#119-F	TTCTGCTATGGCGGGTTC
tHMG-UPL#119-R	GCTGTAACCAAATTCGAAGCA
CrBKT-UPL#159-F	GCTGCTGCAACTGGTTCAC
CrBKT-UPL#159-R	GCACTAGCGGAACTAGCAGAA
CZChYb-UPL#157-F	CGCCCACAAATTACACCATT
CZChYb-UPL#157-R	TCCGAAAAACATACCCCAAG
Kan-UPL#144-F	AGACTAAACTGGCTGACGGAAT
Kan-UPL#144-R	CATCAGGAGTACGGATAAAATGC
alg9-UPL#132-F	CAATCAATGGCCCGTATCAT
alg9-UPL#132-R	TGTCTCAGAAGCACAGTTTGG

### Carotenoid determination

Yeast was freeze-dried by liquid nitrogen and 2.5 Liter Benchtop Freeze Dry System (FreeZone). Freeze-dried yeast was homogenized by MagNA Lyser Green Bead (Roche, Basel, Switzerland) and carotenoids were extracted by acetone. Supernatant was than analyzed by reversed phase HPLC (Jasco PU-2089 Quaternary HPLC equipped with Jasco 870-UV intelligent UV–VIS). Carotenoid was separated in Nomura Chemical Develosil C30-UG Column, 3 µm, ID 4.6 mm x L 250 mm - UG17346250 W (Interlink Scientific Services, Sevenoaks, UK) using mobile phase: A buffer, methanol/MtBE(methyl tert-butyl ether)/Water (81:15:4 vol/vol/vol), and B buffer, methanol/MtBE/Water (7:90:3 vol/vol/vol). The elusion gradient was set as 100%A at 0 min, followed by linear gradient to 100%B at 50 min, and returned to 100%A at 60 min. Carotenoids were identified and quantified by 460 nm absorbance. The accumulation of carotenoids in an engineered strain was estimated as the total amount of carotenoids: Car (mg/g) = 4.69A_440_ x acetone ml/cell weight g. [19].

### Antioxidant capacity assay

After 72 h of culturing in YPG medium (containing 1% yeast extract, 2% peptone, and 2% galactose) at 25 °C, the cells were lyophilized for extraction and analysis. Antioxidant capacity assays of *K. marxianus* wild type (WT) and engineered strains Cz30 were conducted with the ABTS substrate reaction and Trolox Equivalent Antioxidant Capacity (TEAC) assay. ABTS solution was prepared by 4.67 mM ABTS radical cation (Sigma-Aldrich) and 2.45 mM potassiumpersulfate and keep in dark. The assay was performed by mixing ABTS (990 µl) with analytes (10 µl), and the decline of 734 nm absorbance was represented antioxidant ability. Trolox is an analog of vitamin E and TEAC is use trolox equivalent as a benchmark for different antioxidants.

### UV exposure assay

*Kluyveromyces marxianus* WT and Cz30 were tested for survival under UV light exposure. The cell pellets were harvested and exposed to UV light (Viber Lourmat, TFX-20M, 6 × 15 W) for 5, 10, or 20 min, and then dropped into the YPG plate (containing 1% yeast extract, 2% peptone, 2% galactose, and 2% agar) with a series dilution culturing for 72 h at 25 °C.

### Stress tolerance assays

The engineered yeasts were subjected to tolerance tests in YPG medium (containing 1% yeast extract, 2% peptone, and 2% galactose) with initial OD of 1.0 and different initial concentrations of chemicals, including furfural, ethanol, isobutanol, and 10 deacetylbaccatin III. After 24 h, survival tests were conducted in YPG plates with a series dilution culturing at 25 °C for 72 h. The growth rate was measured by 600 nm absorbance at different time points. The growth density experiment was repeated three times and plate assay was performed one time.

### Ethanol production

Cells were grown on YPG medium (containing 1% yeast extract, 2% peptone, and 20% galactose) and the total production of ethanol was analyzed by HPLC (Jasco PU-2089 Quaternary HPLC pump, JASCO International Co., Tokyo, Japan) with thICSep ICE-COREGEL 87H3 Column (Transgenomic, Nebraska, USA) and Shodex RI-101 Refractive Index Detector (ECOM, Praha, Czech Republic). Each experiment was repeated three times.

## Supplementary information

**Additional file 1.** Additional figures.

## Data Availability

The datasets used in this study are available from the corresponding author on request. All data generated or analyzed during this study are included in this published article. There is no additional material.

## Abbreviations

ROS: reactive oxygen species
UV: ultraviolet
PGASO: Promoter-based Gene Assembly and Simultaneous Overexpression
RT-qPCR: quantitative reverse transcription PCR
ABTS: 2,2′-azino-bis (3-ethylbenzothiazoline-6-sulphonic acid)
TEAC: Trolox Equivalent Antioxidant Capacity
As[V]: arsenate
10-DB III: 10-deacetyl baccatin III
YPG: medium containing peptone, yeast extract and galactose
