# Supplementary material for: Characterizing an engineered carotenoid-producing yeast as an anti-stress chassis for building cell factories
Source: Microb Cell Fact. 2019 Sep 10;18:155. doi: 10.1186/s12934-019-1205-y (PMC6737703; doi:10.1186/s12934-019-1205-y)
Supplement: Supplementary file 1 — Additional file 1. Additional figures. [file 12934_2019_1205_MOESM1_ESM.pdf]

Additional file 1

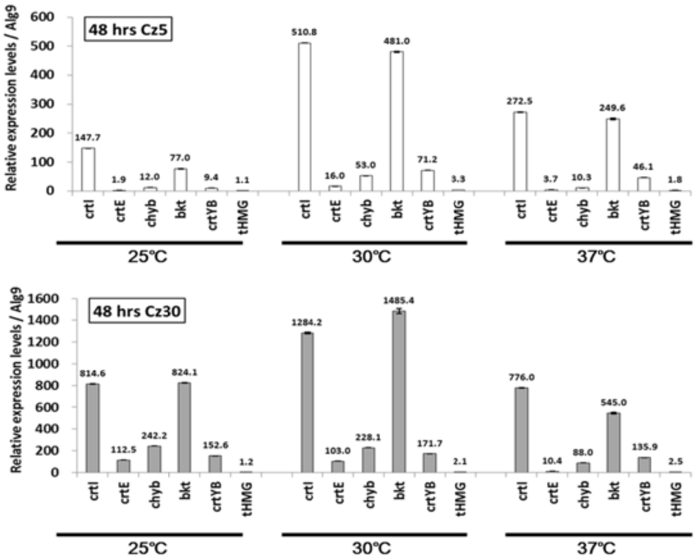

Figure S1. Transcriptional levels of genes in two engineered strains under different growth conditions

The relative gene expression levels in WT, Cz5, and Cz30 at 25°C, 30°C and 37°C compared to the housekeeping gene *Alg9* (alpha-1,2-mannosyltransferase) measured by RT-qPCR. The data represent the mean±SD (n=3)

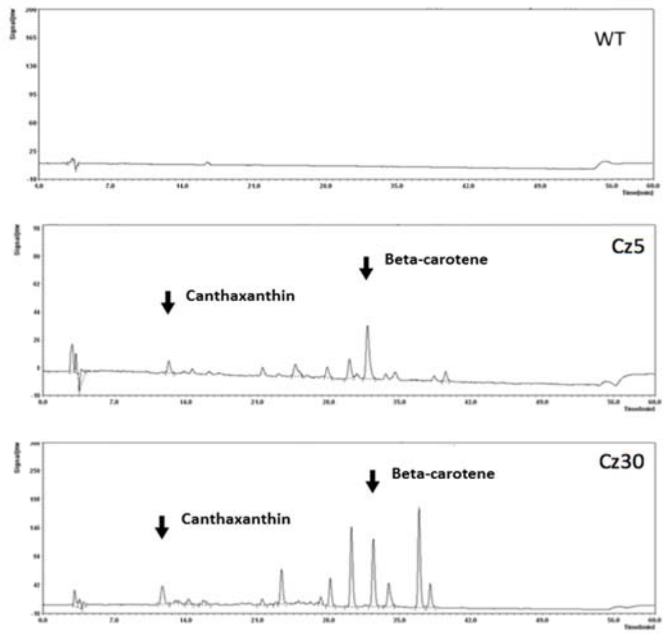

Figure S2. Carotenoids profiles of Cz5 and Cz30

Carotenoids of each strain were measured by HPLC spectrometry assay under UV460 nm. No UV460 nm absorption was found in WT. The carotenoids profile of Cz5 was published in our previous study (Chang et al. 2015) and was used here for the comparison with the profile of Cz30.

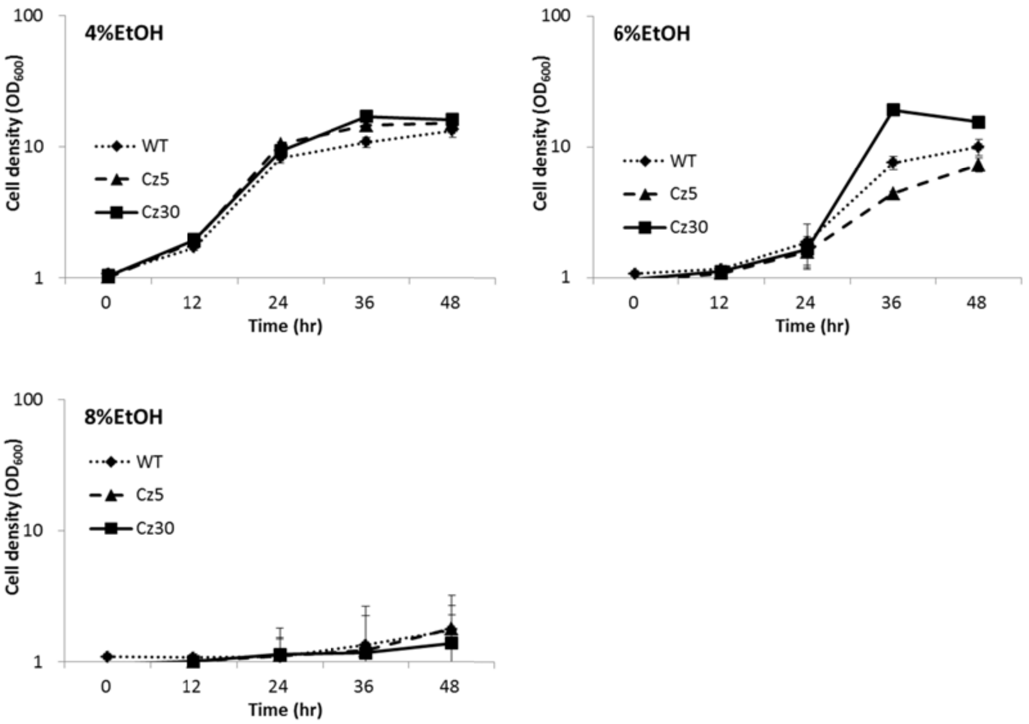

Figure S3. Alcohol tolerance of WT, Cz30 and Cz5 on a log base 10 scale

The growth assay was conducted to evaluate the tolerance of cells to 2%, 4% and 6% ethanol concentrations. The data represent the mean±SD (n=3)

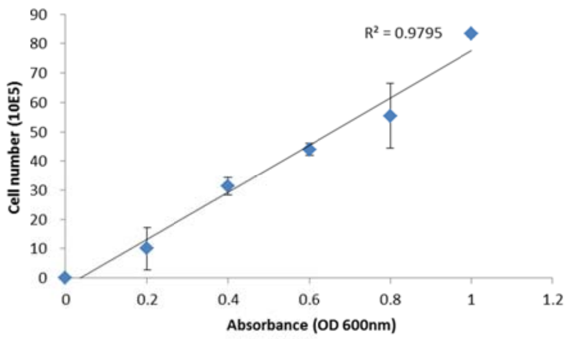

Figure S4. The correlation between colony-forming unit assay and absorbance (OD600nm) assay

The cell concentration of *K. marxianus* was measured by absorbance (OD600nm) assay and by colony forming unit assay. The correlation between the two assays has an R^2 of 0.98.The data represent the mean±SD (n=3).
